# Global dynamic spatiotemporal pattern of seasonal influenza since 2009 influenza pandemic

**DOI:** 10.1186/s40249-019-0618-5

**Published:** 2020-01-03

**Authors:** Zhi-Wei Xu, Zhong-Jie Li, Wen-Biao Hu

**Affiliations:** 1 School of Public Health and Social Work & Institute of Health and Biomedical Innovation, Queensland University of Technology, Brisbane, Australia; 2 Institute of Health and Biomedical Innovation, Queensland University of Technology, Brisbane, Australia; 3 School of Public Health, Faculty of Medicine, University of Queensland, Brisbane, Australia; 4 Division of Infectious Disease, Key Laboratory of Surveillance and Early-warning on Infectious Disease, Chinese Center for Disease Control and Prevention, Beijing, China

**Keywords:** Influenza a, Influenza B, Seasonality, Spatial pattern, Vaccination

## Abstract

**Background:**

Understanding the global spatiotemporal pattern of seasonal influenza is essential for influenza control and prevention. Available data on the updated global spatiotemporal pattern of seasonal influenza are scarce. This study aimed to assess the spatiotemporal pattern of seasonal influenza after the 2009 influenza pandemic.

**Methods:**

Weekly influenza surveillance data in 86 countries from 2010 to 2017 were obtained from FluNet. First, the proportion of influenza A in total influenza viruses (P_A_) was calculated. Second, weekly numbers of influenza positive virus (A and B) were divided by the total number of samples processed to get weekly positive rates of influenza A (RW_A_) and influenza B (RW_B_). Third, the average positive rates of influenza A (R_A_) and influenza B (R_B_) for each country were calculated by averaging RW_A_, and RW_B_ of 52 weeks. A Kruskal-Wallis test was conducted to examine if the year-to-year change in P_A_ in all countries were significant, and a universal kriging method with linear semivariogram model was used to extrapolate R_A_ and R_B_ in all countries.

**Results:**

P_A_ ranged from 0.43 in Zambia to 0.98 in Belarus, and P_A_ in countries with higher income was greater than those countries with lower income. The spatial patterns of high R_B_ were the highest in sub-Saharan Africa, Asia-Pacific region and South America. RW_A_ peaked in early weeks in temperate countries, and the peak of RW_B_ occurred a bit later. There were some temperate countries with non-distinct influenza seasonality (e.g., Mauritius and Maldives) and some tropical/subtropical countries with distinct influenza seasonality (e.g., Chile and South Africa).

**Conclusions:**

Influenza seasonality is not predictable in some temperate countries, and it is distinct in Chile, Argentina and South Africa, implying that the optimal timing for influenza vaccination needs to be chosen with caution in these unpredictable countries.

## Background

Seasonal influenza caused substantial morbidity and mortality worldwide, especially in elderly population and children aged under five years. It is estimated that, from 1999 to 2015, there were 291 243 to 645 832 seasonal influenza-associated respiratory deaths every year globally [1], causing a considerable health burden. For example, Australia witnessed its largest influenza season in 2017 since the 2009 pandemic, posing a substantial burden to primary care and hospitals [2]. Unveiling the global spatial pattern of seasonal influenza is essential for national and international decision making on influenza prevention and control.

Vaccination has been widely recognized as the most effective means of seasonal influenza prevention and can largely ease the burden caused by influenza. Identification of the optimal timing for vaccination is of great importance because vaccine-induced immunity wanes quickly after vaccination [3], and unfolding influenza seasonality is a crucial step for determining optimal vaccination timing. The widespread consensus in the literature is that influenza seasonality pattern is more ascertained in temperate regions/countries, but remains largely unclear and controversial in tropical and subtropical regions/countries [4–6]. It has been suggested that although influenza seasonality in tropics and subtropics is complicated, it might still be possible to group countries into similar zones for tailored and timely vaccination [7].

Prior studies have reported that the epidemiology (e.g., seasonality) of influenza A and influenza B may differ from each other [8–10], and the relative importance of influenza A and influenza B in driving seasonal influenza peak may vary across different countries [11]. For the development of strategic seasonal influenza control programs (e.g., using trivalent vaccines or quadrivalent vaccines), it is essential to assess the proportions of influenza A virus and influenza B virus in seasonal influenza virus.

Global, contemporaneous and comparative analysis of influenza data would help focus resources more effectively on areas/populations that need it most [12, 13]. Previous studies have reported the global spatial and temporal patterns of seasonal influenza up to 2015 [8, 14, 15], but very up-to-date information is not available in existing literature. Our study attempted to characterize the global spatial pattern of seasonal influenza A and B after 2009 influenza pandemic (i.e., from 2010 to 2017), to assess the proportions of influenza A virus and influenza B virus in total influenza virus, and to elucidate the seasonality of seasonal influenza A and B in temperate countries and tropical/subtropical countries. The specific objectives were three-fold: I). what was the proportion of influenza A virus in total influenza positive virus (P_A_) in each country; and whether there were any year-to-year changes in this proportion? II). what were the high risk regions of influenza A and influenza B? and III). what were the global seasonal patterns of influenza A and influenza B?

## Methods

### Data collection

Weekly influenza surveillance data from 2010 to 2017 were collected from FluNet, an online database of WHO Global Influenza Surveillance Network for laboratory-confirmed influenza samples [6, 8]. Detailed information on FluNet can be found in WHO website (http://www.who.int/influenza/gisrs_laboratory/flunet/en/). FluNet data are real-life data. The diagnostic methods may vary widely between countries because of manpower and training issues, but FluNet data are the most widely available data that can be used by WHO surveillance to design the seasonal influenza vaccines. It is not easy practically (if not impossible) to unify the world’s approach to testing for these influenza viruses due to the resource variability and limitations. Thus, FluNet data are still quite valuable despite its limitations. Specifically, the data extracted in this study included the following variables: total number of influenza positive virus, total number of influenza A virus, total number of influenza B virus, and total number of samples processed. Countries with complete data of at least one year from 2010 to 2017 were selected, and in total there were 86 countries included in this study. The detailed influenza information on the countries selected, including time period(s), total number of samples processed, total number of influenza positive virus, total number of influenza A virus, and total number of influenza B virus, is depicted in Additional file 1: Table S1. World Bank categorized all countries into four income groups, including low income, lower middle income, upper middle income, and high income. We collected this information for each included country to assess if P_A_ varied across different income groups (http://blogs.worldbank.org/opendata/new-country-classifications-income-level-2017-2018).

### Data analysis

There were three analytical approaches corresponding to three objectives. First, for each country, total number of influenza A virus was divided by total number of influenza positive virus to get the proportion of influenza A virus in total influenza positive virus (P_A_). Data on 21 countries with complete data from 2010 to 2017 were used to present the year-to-year change in P_A_. The yearly P_A_ data for each country were ratio and were not normally distributed (after normality test), so we conducted a Kruskal-Wallis test to check if the year-to-year changes in P_A_ in all countries were statistically significant. Second, weekly numbers of influenza positive virus for influenza A and influenza B, as well as total number of samples processed, across all years were merged into 52 weeks in each country. Weekly numbers of influenza positive virus (influenza A and influenza B) were divided by the total number of samples processed to get weekly positive rates of influenza A (RW_A_), and influenza B (RW_B_). The average positive rates of influenza A (R_A_), and influenza B (R_B_) for each country were calculated by averaging RW_A_, and RW_B_ of 52 weeks, and a kriging approach was used to extrapolate the average influenza positive rate in all countries globally. Specifically, we used the “universal” kriging method and the “linear” semivariogram model. Universal kriging is a powerful method which simultaneously estimates a trend and used the resulting errors for kriging. The equations for calculating P_A_, RW_A_, RW_B_, R_A_ and R_B_, are presented in Table 1. Third, heat maps were plotted using RW_A_ and RW_B_ to present the seasonal patterns of influenza A and influenza B in temperate countries and tropical/subtropical countries. A cosinor function combined with Poisson regression was used to quantify the peak time and trough time of influenza A and influenza B [16].

**Table 1:** The equations for calculating the indexes

Index	Definition of the index	Equation to calculate the index
P_A_	the proportion of influenza A virus in total influenza positive virus	(total number of influenza A virus) / (total number of influenza positive virus)
RW_A_	weekly positive rate of influenza A	(weekly number of influenza A positive virus) / (total number of samples processed)
RW_B_	weekly positive rate of influenza B	(weekly number of influenza B positive virus) / (total number of samples processed)
R_A_	the average positive rate of influenza A	(RW_Aweek1_ + RW_Aweek2_+… + RW_Aweek52_) / 52
R_B_	the average positive rate of influenza B	(RW_Bweek1_ + RW_Bweek2_+...+RW_Bweek52_) / 52

Spatial mapping and kriging were conducted in ArcGIS 10.5 (ESRI Inc., Redlands, CA, USA), and all other analyses were done in R package 3.4.4 (https://www.r-project.org/).

## Results

### The global spatial pattern of P_A_ and its temporal change

**Fig. 1:**
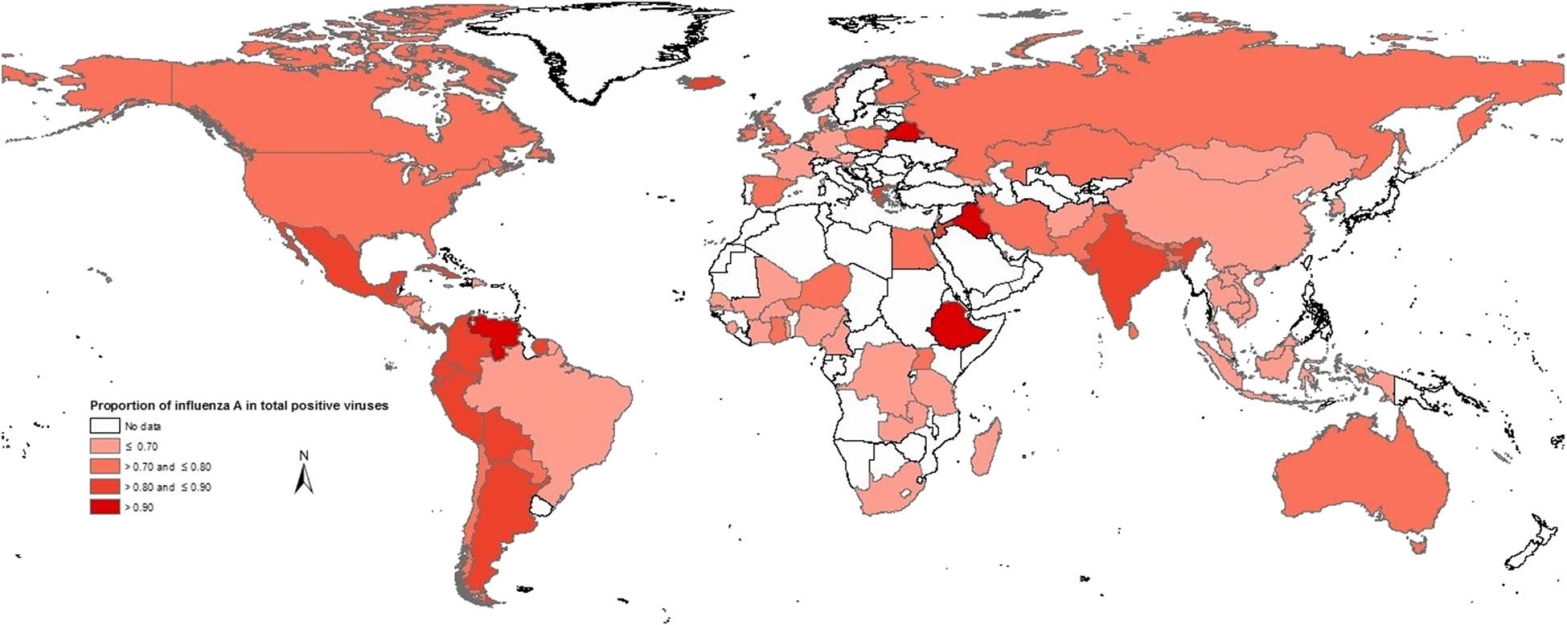
Global pattern of influenza A proportion

**Fig. 2:**
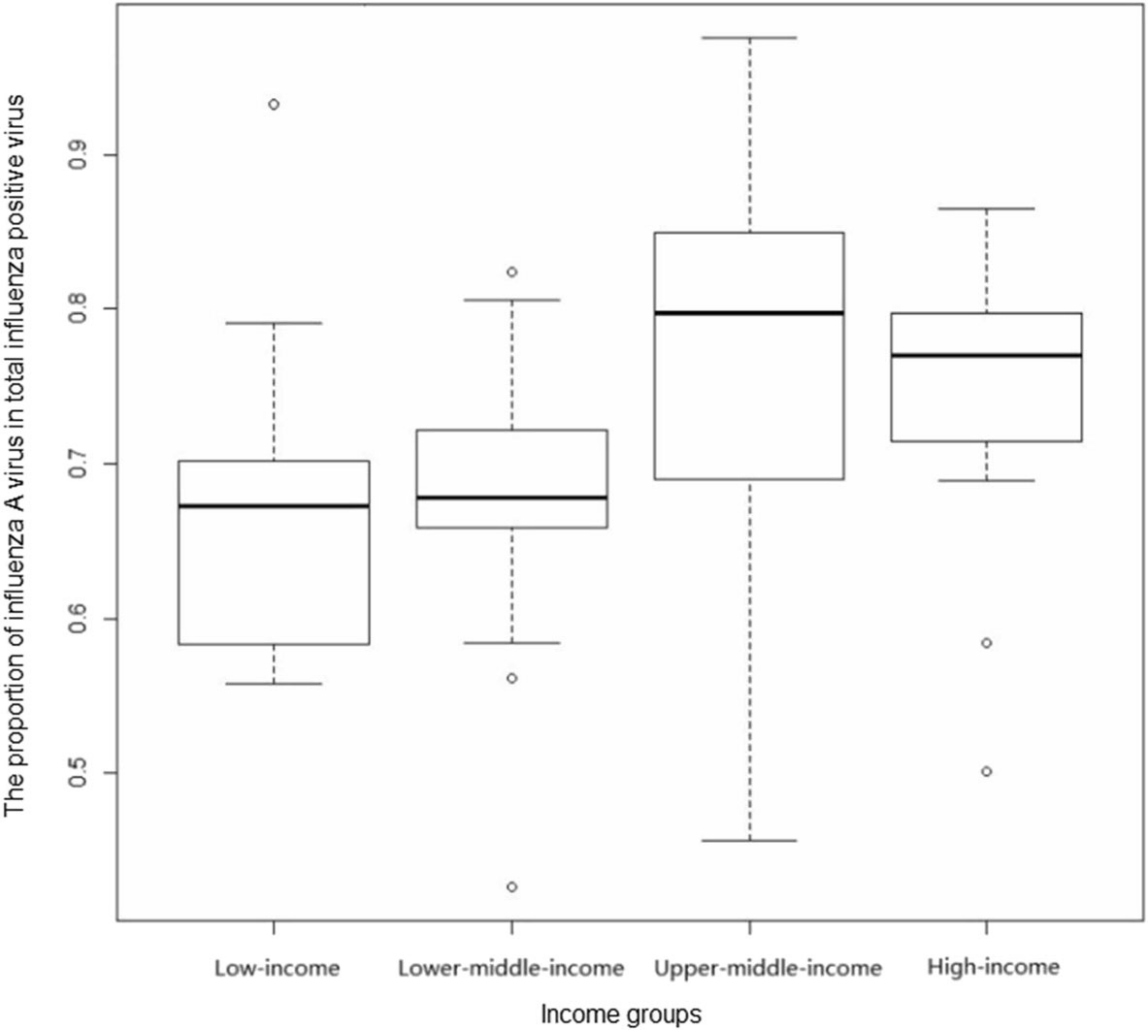
Influenza A proportion in countries from different income groups

**Fig 3:**
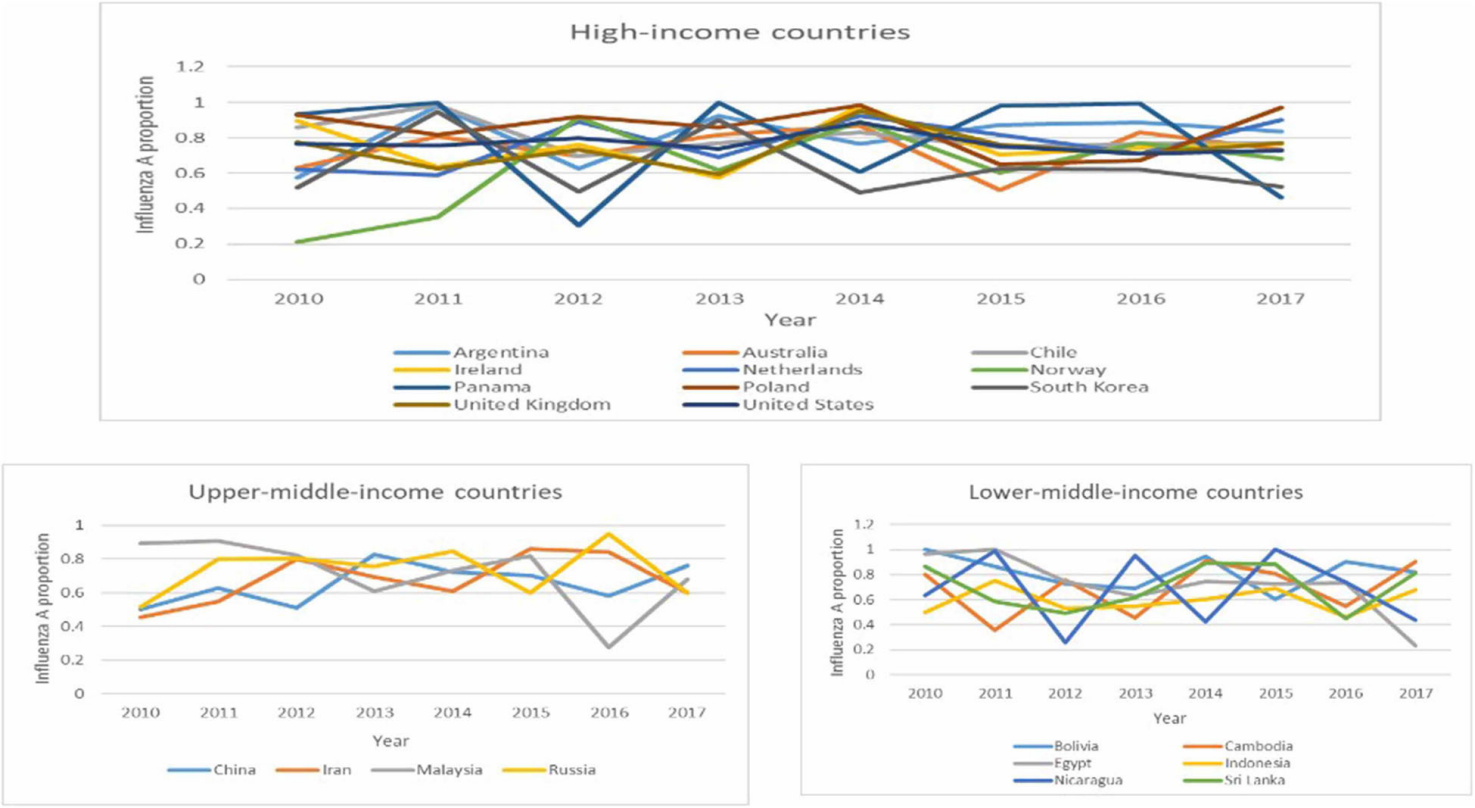
Temporal change in influenza A proportion in 21 countries

The proportion of influenza A virus in total influenza positive virus (P_A_) were illustrated in Fig. 1. The highest P_A_ was in Belarus (upper-middle-income), Ethiopia (low-income), Iraq (upper-middle-income), and Venezuela (upper-middle-income). Specifically, P_A_ was greater than 0.5 in all countries except for Zambia and Lebanon. Fig. 2 shows that P_A_ was higher in high-income and upper-middle-income countries than low-income and lower-middle-income countries (*P* = 0.0015 in the Kruskal-Wallis test), although one of the four countries with the highest P_A_ was a low-income country (i.e., Ethiopia). Fig. 3 shows that there were year-to-year changes in the relative proportions of influenza A virus. The Kruskal-Wallis test indicates that the year-to-year changes globally were not statistically significant (*P* = 0.5271).

### The global spatial patterns of R_A_ and R_B_

**Fig. 4:**
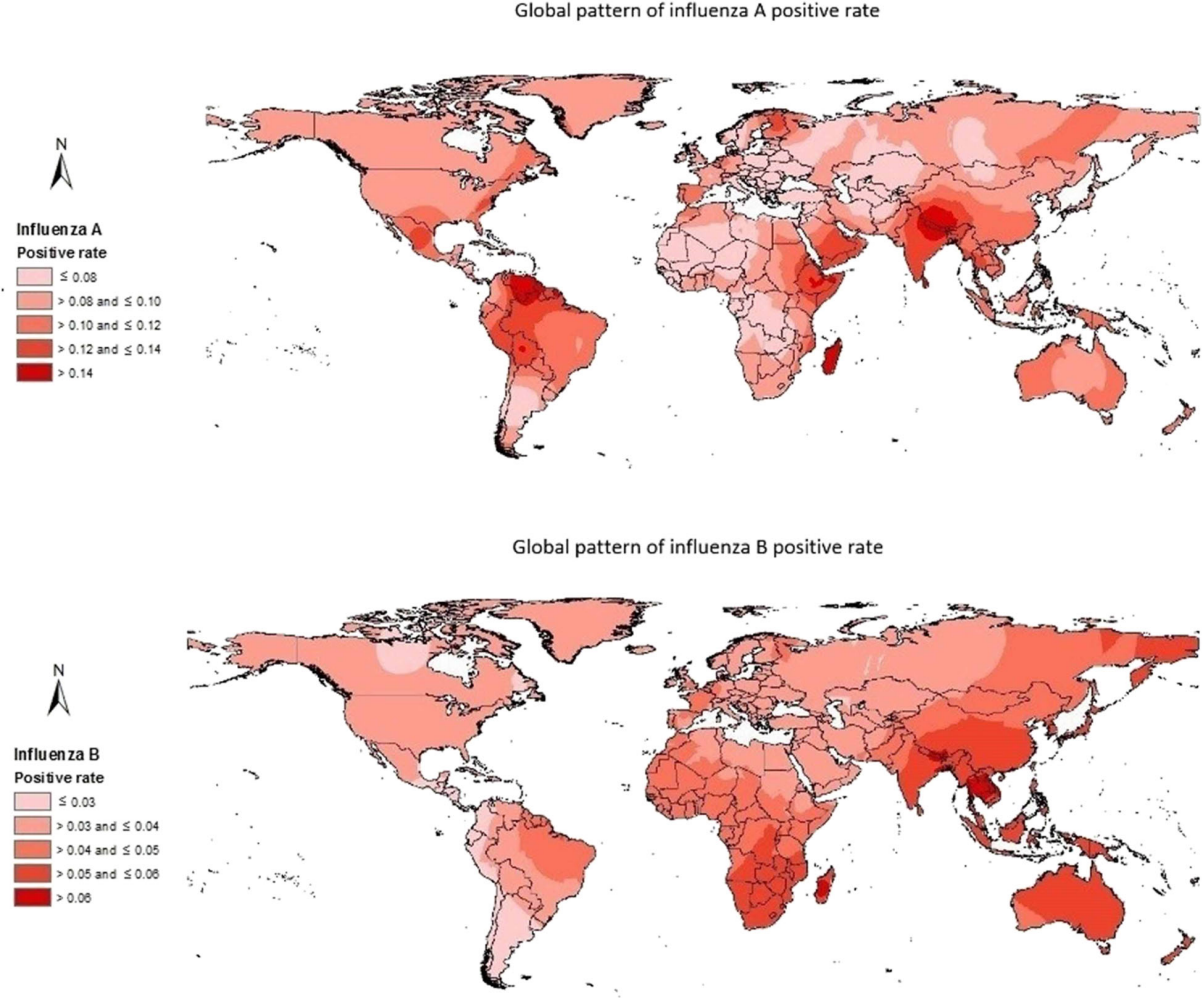
Global pattern of influenza positive rate

The global spatial patterns of R_A_ and R_B_ was shown in Fig. 4. The highest R_A_ distributed in Venezuela, Bolivia, Nepal, Ethiopia and China, and the highest R_B_ distributed in sub-Saharan Africa, Asia-Pacific region and South America.

### The seasonal patterns of influenza a and influenza B

**Fig. 5:**
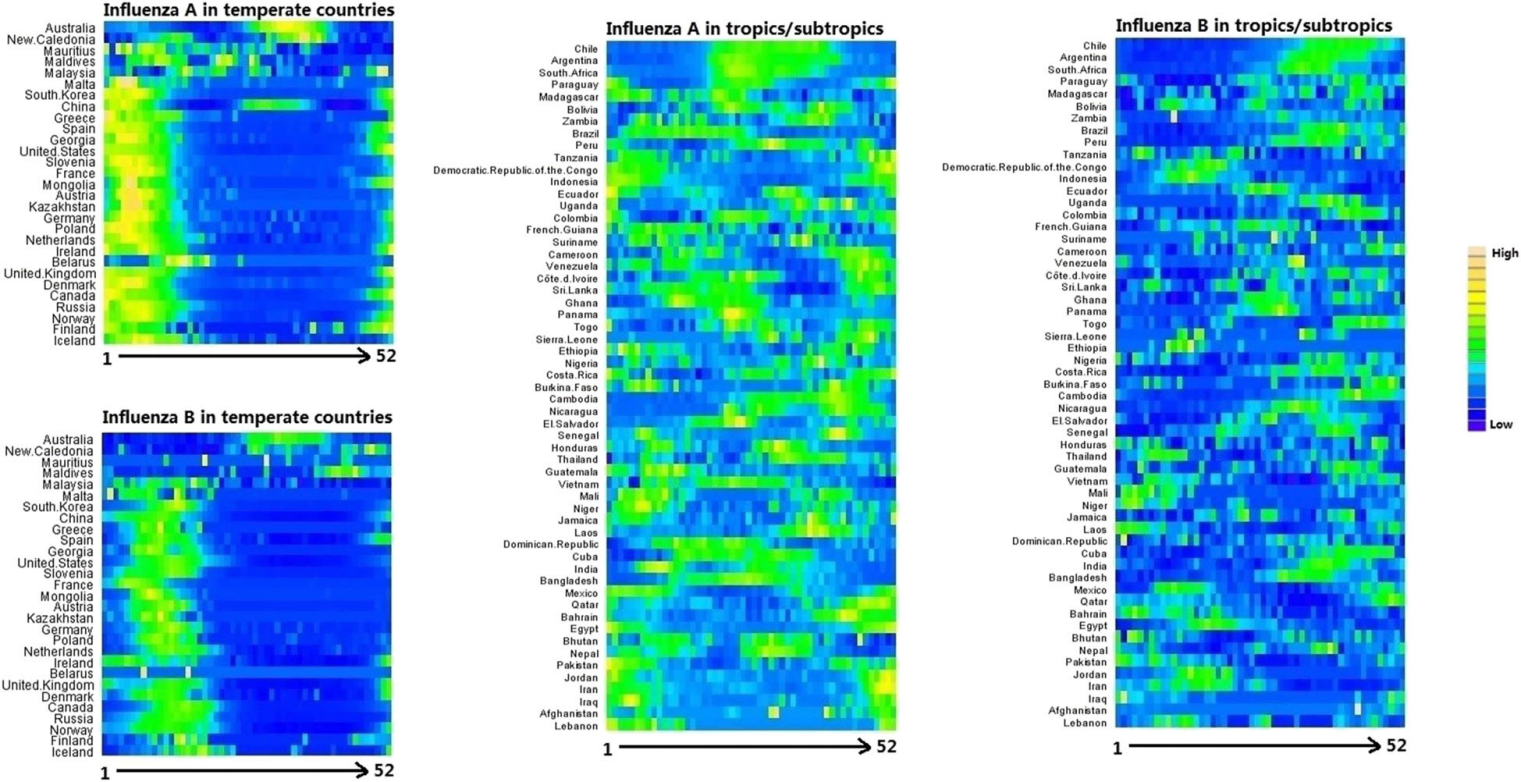
Seasonality of influenza A and B in temperate and tropical/subtropical countries

The seasonal patterns of RW_A_ and RW_B_ in temperate countries and tropical/subtropical countries by latitude were presented in Fig. 5. RW_A_ and RW_B_ peaked in early weeks of each year in most temperate countries except for Australia, New Caledonia, Mauritius, Maldives and Malaysia, and the peak weeks of RW_A_ occurred earlier than influenza RW_B_. For most tropical/subtropical countries, influenza seasonality pattern was diverse (i.e., either having several peaks or occurring all year around). However, influenza seasonality was distinct in Chile, Argentina, and South Africa. The peak time and trough time of influenza in all selected countries quantified by cosinor function are presented in Table 2. The peak time and trough time varied considerably across different countries.

**Table 2:** Peak time and trough time (week) of influenza (listed by latitude)

Country	Peak (A^a^)	Trough (A)	Peak (B^a^)	Trough (B)
Chile	30.5	4.5	40.5	14.5
Argentina	29.3	3.3	37.6	11.6
South Africa	27.9	1.9	35.3	9.3
Australia	33.8	7.8	33.7	7.7
Paraguay	36.7	10.7	39.6	13.6
New Caledonia	33.4	7.4	40.9	14.9
Mauritius	12.4	38.4	28.3	2.3
Madagascar	13.4	39.4	37.3	11.3
Bolivia	30.8	4.8	21.8	47.8
Zambia	31.8	5.8	32.3	6.3
Brazil	15.2	41.2	40	14
Peru	34.3	8.3	37.4	11.4
Tanzania	51.8	26.8	8.2	34.2
Indonesia	2.4	28.4	15.9	41.9
Ecuador	2.6	28.6	24.8	50.8
Democratic Republic of the Congo	2.6	28.6	15.4	41.4
Uganda	40.3	14.3	37.6	11.6
Maldives	16.3	42.3	44.1	18.1
Malaysia	13.4	39.4	12.8	38.8
Colombia	33	7	45.3	19.3
French Guiana	9.2	35.2	22.8	48.8
Suriname	28.4	2.4	30.7	4.7
Cameroon	50.4	24.4	44.1	18.1
Venezuela	1.9	27.9	32.3	6.3
Côte d’Ivoire	25.1	51.1	37.8	11.8
Sri Lanka	10	36	4.7	30.7
Ghana	18.7	44.7	34.8	8.8
Panama	24.2	50.2	28.5	2.5
Togo	34	8	46.7	20.7
Sierra Leone	40	14	25.4	51.4
Ethiopia	52.9	26.9	12.8	38.8
Nigeria	39	13	47	21
Costa Rica	45	19	37.4	11.4
Burkina Faso	51.7	25.7	50	24
Cambodia	37	11	42.9	16.9
Nicaragua	36.9	10.9	33.4	7.4
El Salvador	22.9	48.9	34.2	8.2
Senegal	47.4	21.4	31.1	5.1
Honduras	24.8	50.8	51.7	25.7
Thailand	37.4	11.4	5.6	31.6
Guatemala	9.4	35.4	40.8	14.8
Vietnam	22.7	48.7	4.9	30.9
Mali	3	29	4.3	30.3
Niger	2.8	28.8	5.4	31.4
Jamaica	47.1	21.1	31	5
Laos	38.8	12.8	2.1	28.1
Deminican Republic	18.2	44.2	31	5
Cuba	25.4	51.4	40.8	14.8
India	17.5	43.5	39.9	13.9
Bangladesh	25.3	51.3	34.7	8.7
Mexico	2.1	28.1	6.8	32.8
Qatar	49.6	23.6	4.6	30.6
Bahrain	45.2	19.2	10.1	36.1
Egypt	51.1	25.1	19.8	45.8
Bhutan	24.8	50.8	51.4	25.4
Nepal	26.7	52.7	41.1	15.1
Pakistan	51.6	25.6	5.5	31.5
Jordan	52.7	26.7	14.9	40.9
Iran	1	27	14.3	40.3
Iraq	1.6	27.6	2.7	28.7
Afghanistan	43.4	17.4	46.3	20.3
Lebanon	4.4	30.4	9.7	35.7
Malta	5.7	31.7	8.7	34.7
South Korea	4.8	30.8	12.1	38.1
China	4.2	30.2	8.4	34.4
Greece	7.1	33.1	10.5	36.5
Spain	4.1	30.1	9.1	35.1
Georgia	5.7	31.7	10.8	36.8
United States	4.1	30.1	12.2	38.2
Slovenia	5.9	31.9	10.7	36.7
France	6.2	32.2	7.2	33.2
Mongolia	4.5	30.5	10	36
Austria	7	33	10.8	36.8
Kazakhstan	6.5	32.5	9.6	35.6
Germany	6.7	32.7	12.2	38.2
Poland	6	32	10.7	36.7
Netherlands	6.7	32.7	12.7	38.7
Ireland	5.8	31.8	8	34
Belarus	14.3	40.3	12.4	38.4
United Kingdom	5.4	31.4	9.1	35.1
Denmark	5.4	31.4	9.3	35.3
Canada	4	30	14	40
Russia	7.2	33.2	12.6	38.6
Norway	5.8	31.8	11.2	37.2
Finland	52.8	26.8	9.5	35.5
Iceland	8.3	34.3	15.4	41.4

^a^A: influenza A; B: influenza B

The seasonal patterns of RW_A_ and RW_B_ in 21 countries with complete data from 2010 to 2017 were shown in Additional file 2: Figure S1. Influenza seasonality in the two tropical/subtropical countries, i.e., Chile and Argentina, was steadily distinct across different years.

## Discussion

This study used data up to 2017 and kriging approach to unravel the updated global spatial pattern of seasonal influenza. It quantified the proportion of influenza A virus in total influenza virus and modeled the peak times of influenza A and influenza B in each country from 2010 to 2017. Three findings are note-worthy. First, the highest P_A_ was observed in Belarus, Ethiopia, Iraq, and Venezuela, and P_A_ changed from year to year. Second, for influenza B, high risk regions distributed in sub-Saharan Africa, Asia-Pacific region and South America. Third, influenza seasonality was distinct in most temperate countries but there were some exceptions (e.g., Mauritius and Maldives), and influenza seasonality was surprisingly distinct in some tropical/subtropical countries, including Chile, Argentina and South Africa.

Unsurprisingly, we observed that influenza A was the dominant subtype in almost all countries (except for Zambia and Lebanon). Notwithstanding, we found that the proportion of influenza B was greater than the proportion of influenza A in certain years in Malaysia, Nicaragua, Panama, Egypt, and Norway. Iuliano et al. estimated the global burden of influenza-associated respiratory deaths and reported that the highest mortality rate was found in sub-Saharan Africa and southeast Asia, and among those who are aged 75 years or older [1]. The high positive rate of influenza B that we observed in sub-Saharan Africa suggested that not only mortality but also morbidity in this region were high, calling for more influenza prevention resources to be allocated to this socioeconomically-disadvantaged region. Previous studies have also highlighted the necessity of building comprehensive influenza surveillance system in sub-Saharan Africa [17–19]. Regarding the influenza situation in Asia-Pacific region, some countries such as Australia and China had a high influenza positive rate (finding of the present study) but low/moderate mortality rate [1], implying a relatively good healthcare system in these countries but also suggesting a strong need to identify the national, regional, and local optimal vaccination timing for cost-effective influenza prevention (especially for China as it has wide latitude spans) [10], and to build up influenza early warning system which gives warning in a timely manner (e.g., internet-based early warning tools incorporating information collected through traditional surveillance system) [20]. Our prior works have suggested that early warning of infectious diseases using data from search engine (e.g., Google and Baidu) may shed some new light on infectious disease control [21, 22]. The constrains and barriers for influenza control and prevention in Asia-Pacific region are multifaceted (e.g., logistic and resourcing issues) [23], and preventing people in this region from influenza attacks requires concerted efforts from policy makers, public health officials, healthcare workers, and scientists.

The noticeable change of influenza seasonality in the included countries that we observed in this study, to some extent, indicates that there is no one-size-suits-all vaccination timing for tropical/subtropical countries and some temperate countries. Grouping tropical/subtropical countries into several zones for influenza vaccination might need much more detailed works (e.g., identifying the fundamental determinants behind the year-to-year change in seasonality etc.). A prior study investigating the global environmental drivers found that absolute humidity and temperature drive the outbreaks of seasonal influenza [24], and the season of influenza in Vietnam has been found coinciding with the rainy seasons [4], implying that future endeavors aiming to look at the relationships between climatic factors and influenza season in a regional or local scale in tropical and subtropical countries are warranted.

This study has two strengths. First, it unfolded the global spatial pattern of influenza positive rate, which may aid policy making in influenza control and prevention. Second, it identified some temperate countries with non-distinct influenza seasonality and some tropical/subtropical countries with distinct influenza seasonality. Five limitations of this study need to be acknowledged. First, there was sampling bias due to overall bias of case data being reported between different countries. Second, the time periods for all selected countries were not consistent, although they were all within the range of 2010 to 2017. It would benefit the influenza surveillance a lot if some countries with data covering a short period of time (e.g., Mauritius) had more resources injection. Third, the country-level data restricted us to explore the socioecological drivers of influenza seasonality. Fourth, different subtypes of influenza A have different and complex transmission routes, and the results of this study only present a global figure on all influenza A subtypes. Fifth, there were large amount of missing data on the subtypes of influenza A in the FluNet data, which restricted us to distinguish and study the patterns of different influenza viruses.

## Conclusions

Influenza control and prevention attention can predominantly be paid to influenza A in countries such as Venezuela. Sub-Saharan Africa needs more influenza control resources, and efficient influenza prevention programs in Asia-Pacific region call for state-of-the-art internet-based influenza early warning system incorporating traditional surveillance data. Future attempts using spatiotemporal approaches to explore the drivers (e.g., socio-ecological factors) behind the influenza seasonality of tropical and subtropical countries are warranted.

## Supplementary information

**Additional file 1: Table S1.** Detailed information on influenza in the selected countries (listed by latitude).

**Additional file 2: Figure S1.** Seasonality patterns of influenza A and influenza B in countries of temperate climate and of tropical or subtropical climate, from 2010 to 2017.

## Data Availability

The data used in this study are publicly available data, and can be accessed from WHO FluNet (https://www.who.int/influenza/gisrs_laboratory/flunet/en/).

## Abbreviations

P_A_: The proportion of influenza A virus in total influenza positive virus;
R_A_: The average positive rate of influenza A
R_B_: The average positive rate of influenza B
RW_A_: Weekly positive rate of influenza A
RW_B_: Weekly positive rate of influenza B
