# Supplementary material for: Global dynamic spatiotemporal pattern of seasonal influenza since 2009 influenza pandemic
Source: Infect Dis Poverty. 2020 Jan 3;9:2. doi: 10.1186/s40249-019-0618-5 (PMC6942408; doi:10.1186/s40249-019-0618-5)
Supplement: Supplementary file 1 — Additional file 1: Table S1. Detailed information on influenza in the selected countries (listed by latitude). [file 40249_2019_618_MOESM1_ESM.docx]

**Table S1**. Detailed information on influenza in the selected countries (listed by latitude)

| Country | Time period(s) | Processed | A | B | Influenza |
| --- | --- | --- | --- | --- | --- |
| Chile | 2010–2017 | 337 043 | 16 017 | 4236 | 20 253 |
| Argentina | 2010–2017 | 572 715 | 27 105 | 5320 | 32 425 |
| South Africa | 2010–2014 | 39 049 | 4413 | 1946 | 6359 |
| Australia | 2010–2017 | 221 724 | 25 489 | 8909 | 34 398 |
| Paraguay | 2010–2014, 2016-2017 | 37 342 | 4360 | 1358 | 5718 |
| New Caledonia | 2010–2015, 2017 | 5351 | 601 | 427 | 1028 |
| Mauritius | 2013, 2016 | 2708 | 290 | 11 | 301 |
| Madagascar | 2010–2012, 2014, 2016–2017 | 7704 | 1819 | 1112 | 2931 |
| Bolivia | 2010–2017 | 33 997 | 6862 | 1468 | 8330 |
| Zambia | 2011–2014, 2016–2017 | 8444 | 301 | 404 | 705 |
| Brazil | 2017 | 18 527 | 1847 | 1132 | 2979 |
| Peru | 2010–2015, 2017 | 28 774 | 4374 | 922 | 5296 |
| Tanzania | 2010–2011, 2013–2016 | 10 140 | 836 | 363 | 1199 |
| Indonesia | 2010–2017 | 36 029 | 4554 | 3245 | 7799 |
| Ecuador | 2011–2014, 2016–2017 | 32 368 | 3506 | 605 | 4111 |
| Democratic Republic of the Congo | 2010, 2012–2014, 2017 | 9737 | 529 | 384 | 913 |
| Uganda | 2011 | 76 906 | 7602 | 2037 | 9609 |
| Maldives | 2016–2017 | 1897 | 379 | 91 | 470 |
| Malaysia | 2010–2017 | 24 017 | 1015 | 462 | 1477 |
| Colombia | 2010–2012, 2014, 2016–2017 | 59 110 | 3728 | 495 | 4223 |
| French Guiana | 2010–2017 | 2877 | 655 | 373 | 1028 |
| Suriname | 2016–2017 | 919 | 66 | 11 | 77 |
| Cameroon | 2010–2015 | 14 156 | 1548 | 1211 | 2759 |
| Venezuela | 2011, 2014, 2016 | 10 898 | 3322 | 85 | 3407 |
| Côte d'Ivoire | 2012–2017 | 12 878 | 1203 | 600 | 1803 |
| Sri Lanka | 2010–2017 | 33 711 | 5572 | 1947 | 7519 |
| Ghana | 2010–2016 | 23 542 | 2700 | 1069 | 3769 |
| Panama | 2010–2017 | 20 926 | 1593 | 359 | 1952 |
| Togo | 2011–2013, 2016–2017 | 3444 | 414 | 309 | 723 |
| Sierra Leone | 2012–2013 | 1215 | 49 | 35 | 84 |
| Ethiopia | 2010, 2012, 2016 | 2082 | 404 | 29 | 433 |
| Nigeria | 2011–2012, 2014–2017 | 6947 | 468 | 229 | 697 |
| Costa Rica | 2010–2014, 2016–2017 | 28 512 | 3245 | 867 | 4112 |
| Burkina Faso | 2012–2013 | 1087 | 82 | 65 | 147 |
| Cambodia | 2010–2017 | 23 139 | 2539 | 1538 | 4077 |
| Nicaragua | 2010–2017 | 38 303 | 3553 | 1683 | 5236 |
| El Salvador | 2011–2017 | 16 104 | 1100 | 434 | 1534 |
| Senegal | 2010–2014, 2017 | 29 022 | 3488 | 1824 | 5312 |
| Honduras | 2010–2011, 2016 | 5958 | 577 | 216 | 793 |
| Thailand | 2013–2017 | 17 010 | 2770 | 1456 | 4226 |
| Guatemala | 2011, 2013, 2016 | 5696 | 458 | 83 | 541 |
| Vietnam | 2011–2017 | 26 467 | 3540 | 1695 | 5235 |
| Mali | 2010, 2012, 2016 | 8482 | 259 | 48 | 307 |

Processed: total number of samples processed; influenza: total number of influenza positive viruses; A: total number of influenza A virus; B: total number of influenza B virus.

**Table S1**. Detailed information on influenza in the selected countries (listed by latitude) (continued)

| Country | Time period(s) | Processed | A | B | Influenza |
| --- | --- | --- | --- | --- | --- |
| Niger | 2010–2017 | 5076 | 355 | 151 | 506 |
| Jamaica | 2010, 2012–2015, 2017 | 3419 | 211 | 175 | 386 |
| Laos | 2011–2015, 2017 | 17 877 | 1626 | 948 | 2574 |
| Deminican Republic | 2011–2013, 2016–2017 | 8501 | 1052 | 465 | 1517 |
| Cuba | 2010–2011, 2013–2014, 2016 | 24 687 | 2700 | 541 | 3241 |
| India | 2011–2016 | 73 291 | 10 560 | 2536 | 13 096 |
| Bangladesh | 2010–2013, 2015 | 20 265 | 2072 | 824 | 2896 |
| Mexico | 2010–2014, 2016–2017 | 154 569 | 33 826 | 6544 | 40 370 |
| Qatar | 2012–2017 | 95 471 | 15 852 | 4733 | 20 585 |
| Bahrain | 2012–2017 | 4370 | 948 | 219 | 1167 |
| Egypt | 2010–2017 | 63 801 | 9976 | 2713 | 12 689 |
| Bhutan | 2013–2017 | 6010 | 1015 | 401 | 1416 |
| Nepal | 2012–2017 | 18 768 | 5590 | 2273 | 7863 |
| Pakistan | 2010–2013, 2016 | 10 392 | 1685 | 522 | 2207 |
| Jordan | 2012–2014, 2016–2017 | 8375 | 939 | 196 | 1135 |
| Iran | 2010–2017 | 69 632 | 7139 | 2679 | 9818 |
| Iraq | 2010–2017 | 10 006 | 1768 | 118 | 1886 |
| Afghanistan | 2016–2017 | 4808 | 245 | 119 | 364 |
| Lebanon | 2015–2017 | 3028 | 207 | 246 | 453 |
| Malta | 2013–2014 | 1762 | 257 | 95 | 352 |
| South Korea | 2010–2017 | 98 444 | 10 107 | 6643 | 16 750 |
| China | 2010–2017 | 3 026 670 | 313 510 | 158 104 | 471 614 |
| Greece | 2010, 2012–2013 | 7955 | 1745 | 272 | 2017 |
| Spain | 2010, 2012–2017 | 113 788 | 39 842 | 11 887 | 51 729 |
| Georgia | 2011–2013, 2015–2017 | 6389 | 1230 | 662 | 1892 |
| United States | 2010–2017 | 4 640 031 | 509 446 | 162 229 | 671 675 |
| Slovenia | 2011–2017 | 93 496 | 11 140 | 4777 | 15 917 |
| France | 2013 | 82 492 | 5355 | 5336 | 10 691 |
| Mongolia | 2010–2015 | 24 387 | 2086 | 1018 | 3104 |
| Austria | 2011–2013 | 20 015 | 3081 | 1389 | 4470 |
| Kazakhstan | 2011–2013 | 14 048 | 832 | 287 | 1119 |
| Germany | 2012–2016 | 17 421 | 4291 | 1922 | 6213 |
| Poland | 2010–2017 | 34 617 | 7324 | 1834 | 9158 |
| Netherlands | 2010–2017 | 36 861 | 11 934 | 3359 | 15 293 |
| Ireland | 2010–2017 | 89 427 | 9382 | 3184 | 12 566 |
| Belarus | 2012 | 2467 | 79 | 2 | 81 |
| United Kingdom | 2010–2017 | 357 791 | 54 828 | 20 054 | 74 882 |
| Denmark | 2010, 2012–2014, 2017 | 76 906 | 7602 | 2037 | 9639 |
| Canada | 2010, 2013–2017 | 1 268 573 | 151 504 | 38 843 | 190 347 |
| Russia | 2010–2017 | 936 296 | 105 132 | 30 285 | 135 417 |
| Norway | 2010–2017 | 609 521 | 58 398 | 25 951 | 84 349 |
| Finland | 2015–2017 | 1373 | 429 | 112 | 541 |
| Iceland | 2012–2017 | 8026 | 1226 | 230 | 1456 |

Processed: total number of samples processed; influenza: total number of influenza positive viruses; A: total number of influenza A virus; B: total number of influenza B virus.
