# Supplementary material for: Global dynamic spatiotemporal pattern of seasonal influenza since 2009 influenza pandemic
Source: Infect Dis Poverty. 2020 Jan 3;9:2. doi: 10.1186/s40249-019-0618-5 (PMC6942408; doi:10.1186/s40249-019-0618-5)

**Seasonality patterns of influenza A and influenza B in countries of temperate climate and of tropical or subtropical climate, from 2010 to 2017**

**Part one: Countries of temperate climate**


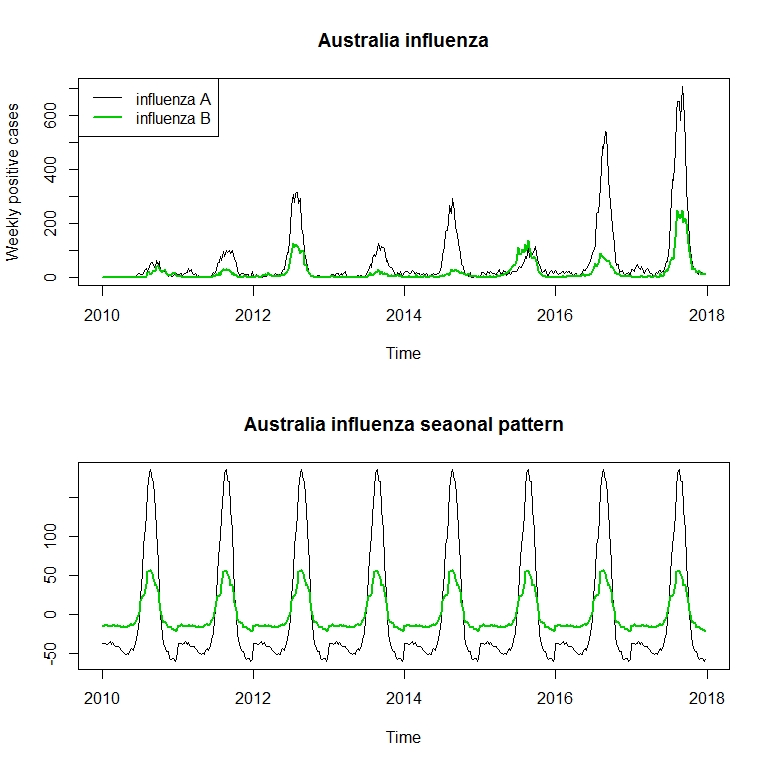


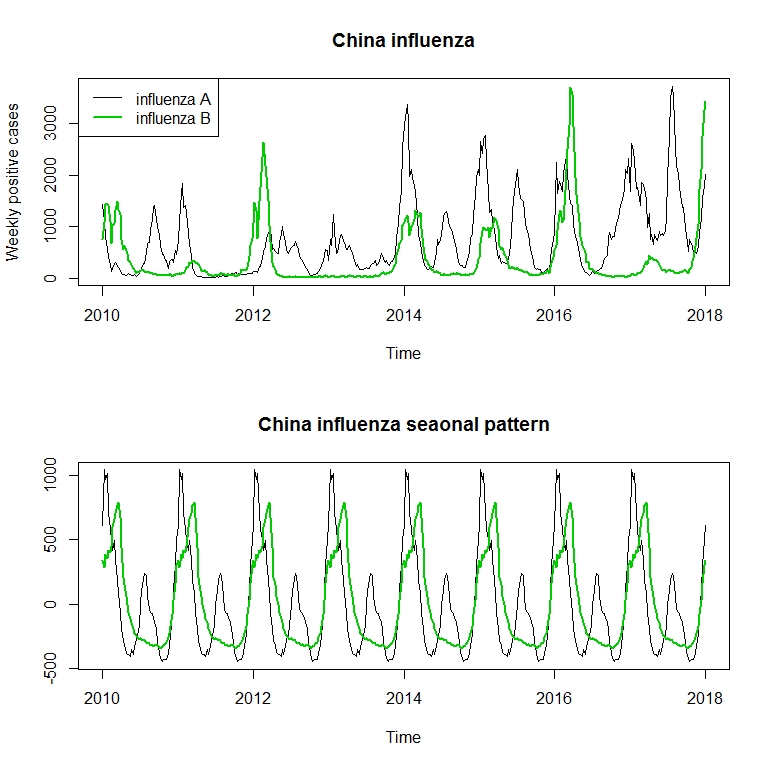


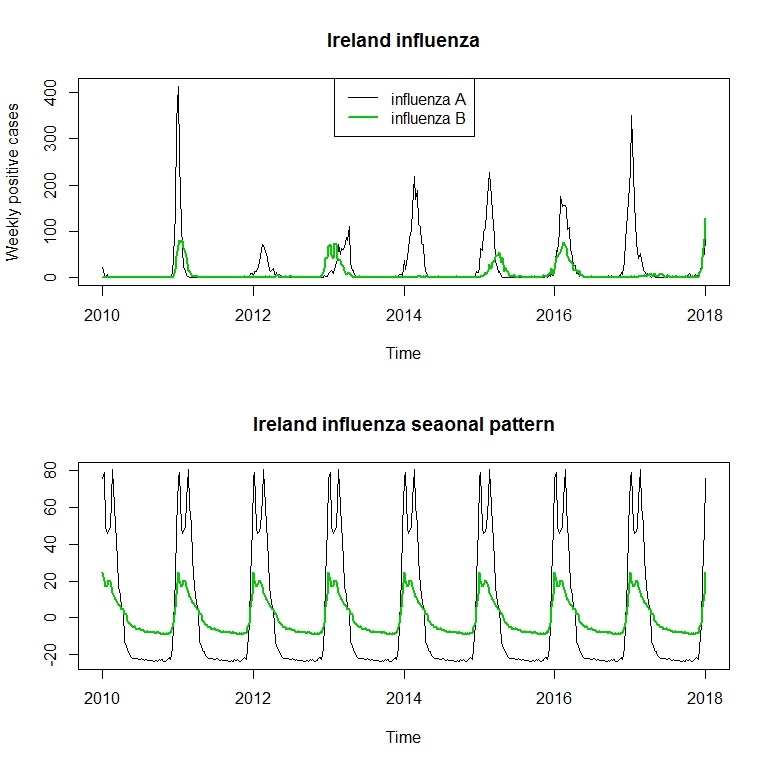


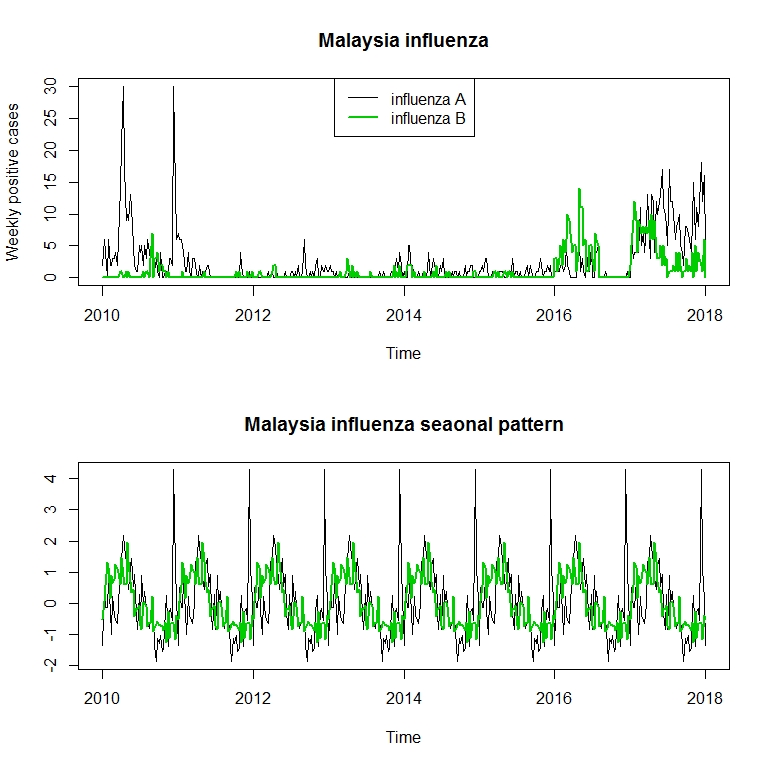


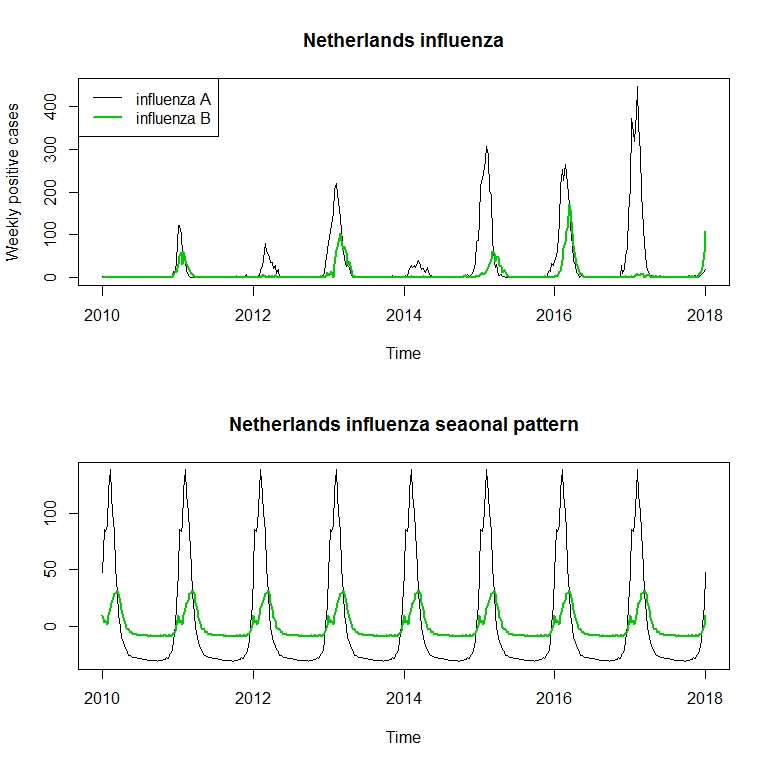


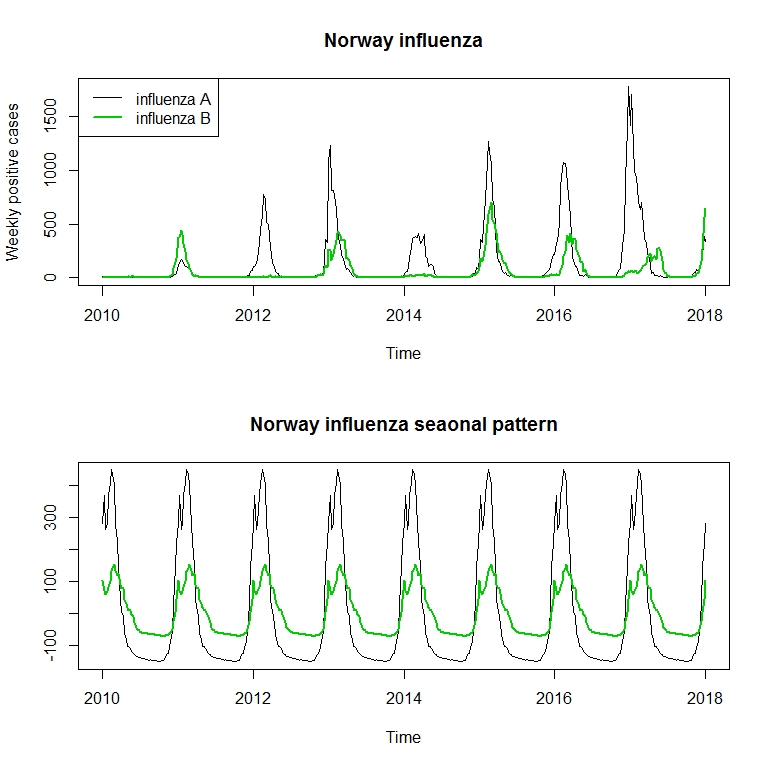


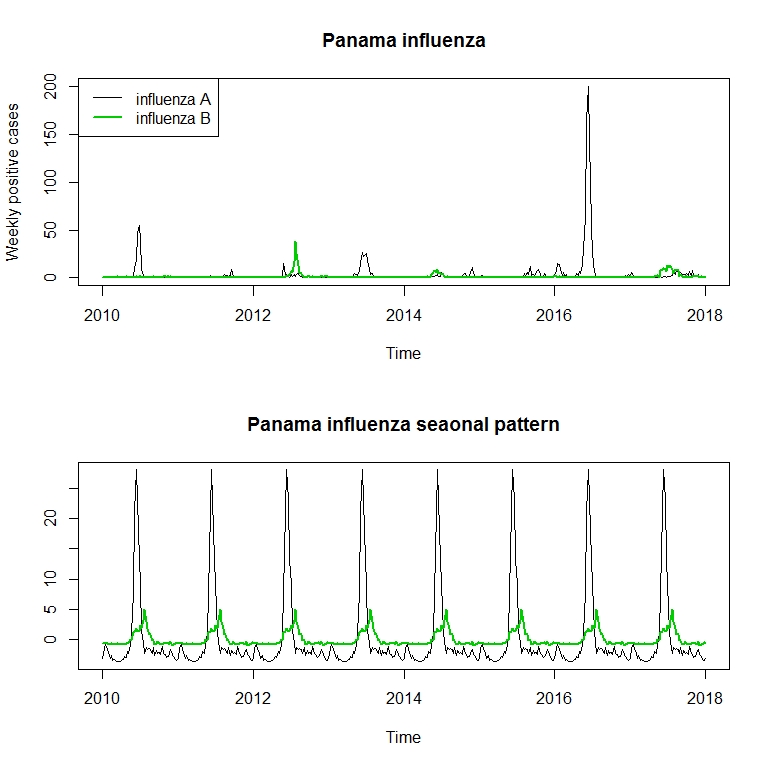


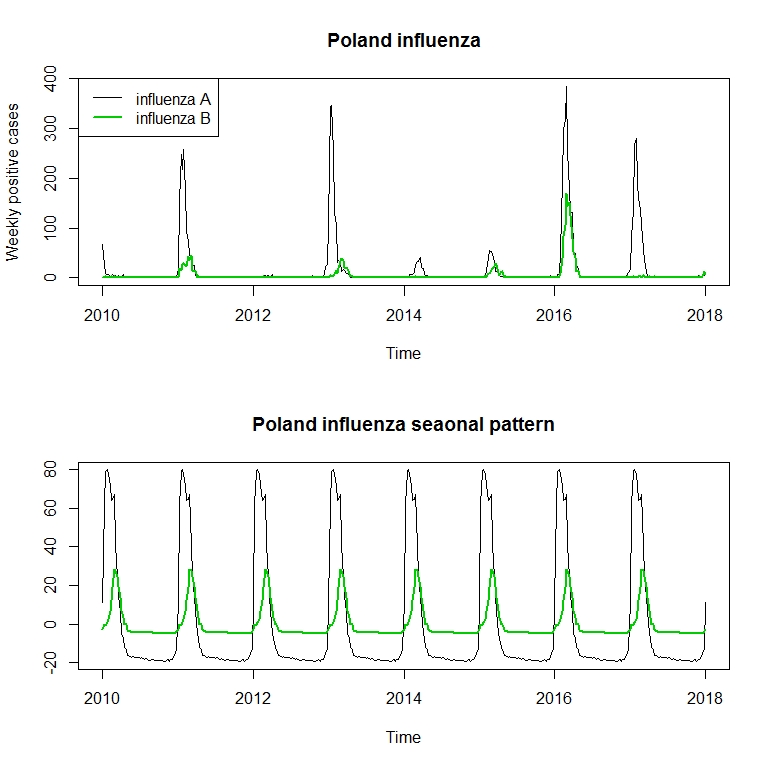


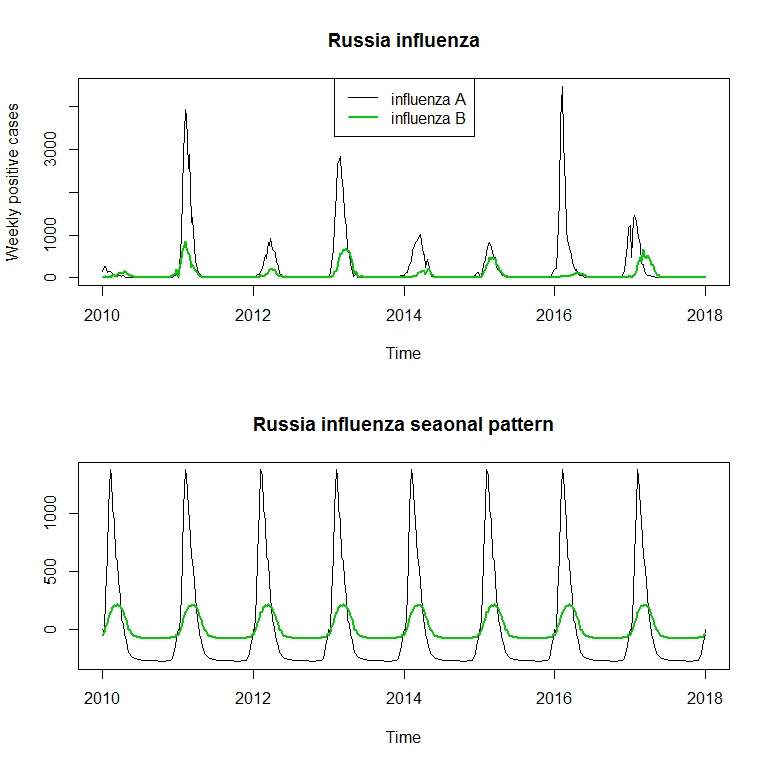


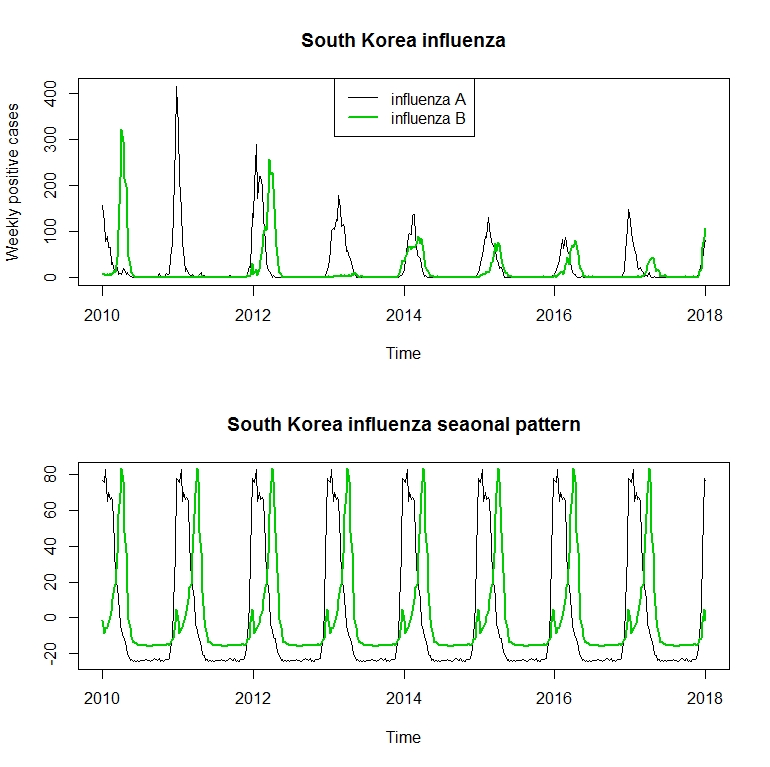


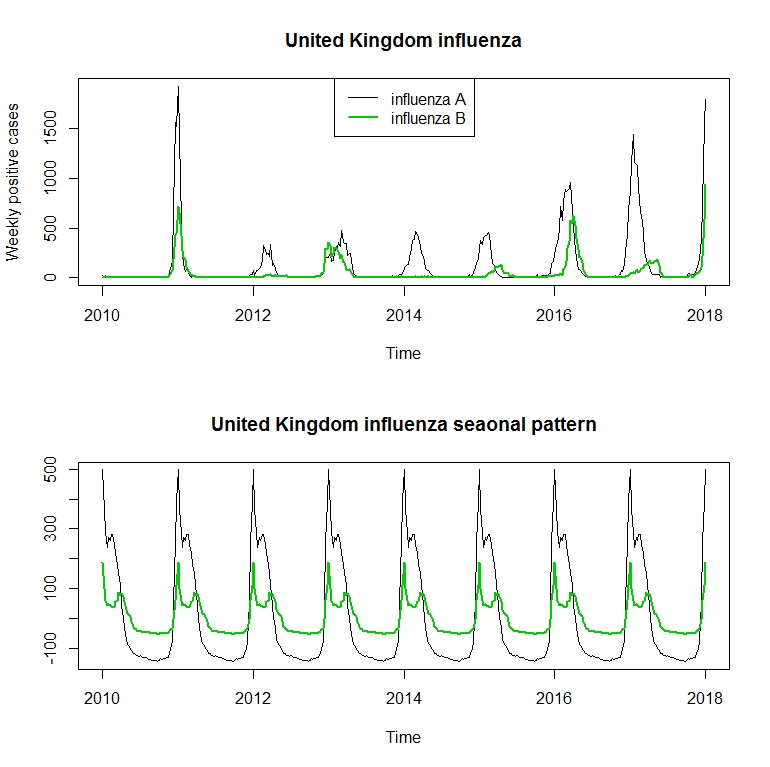


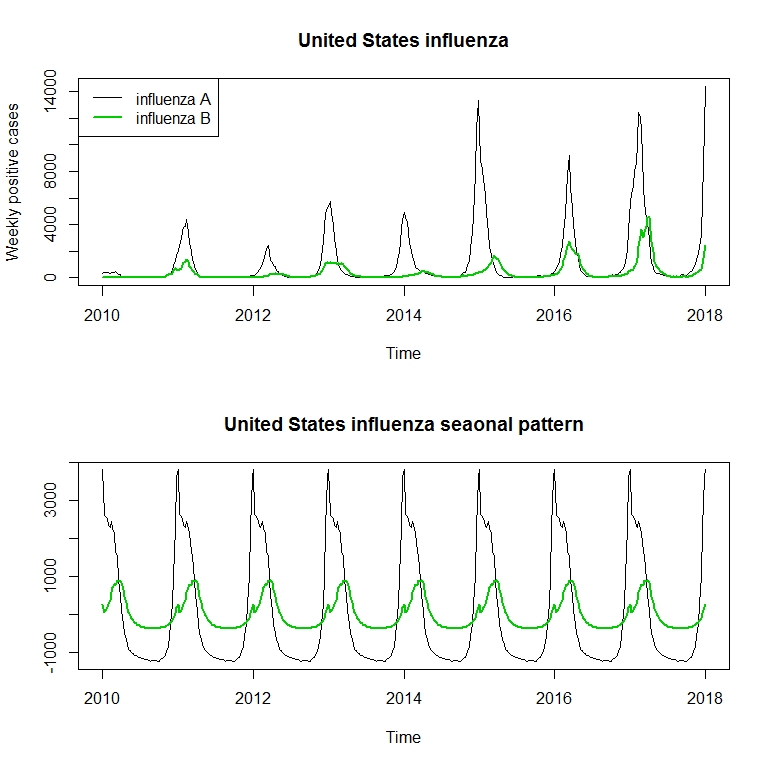


**Part two: Countries of tropical or subtropical climate**


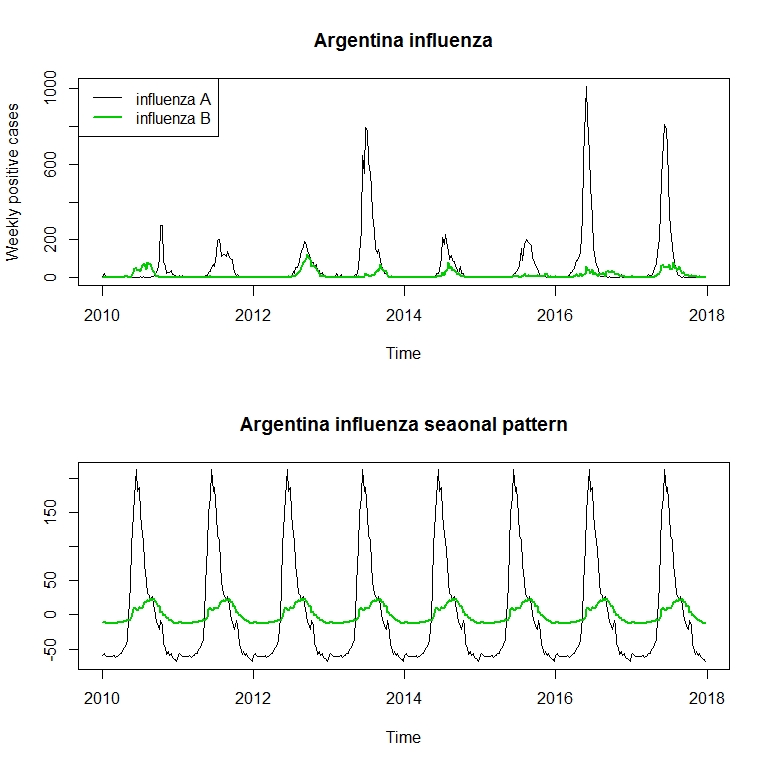


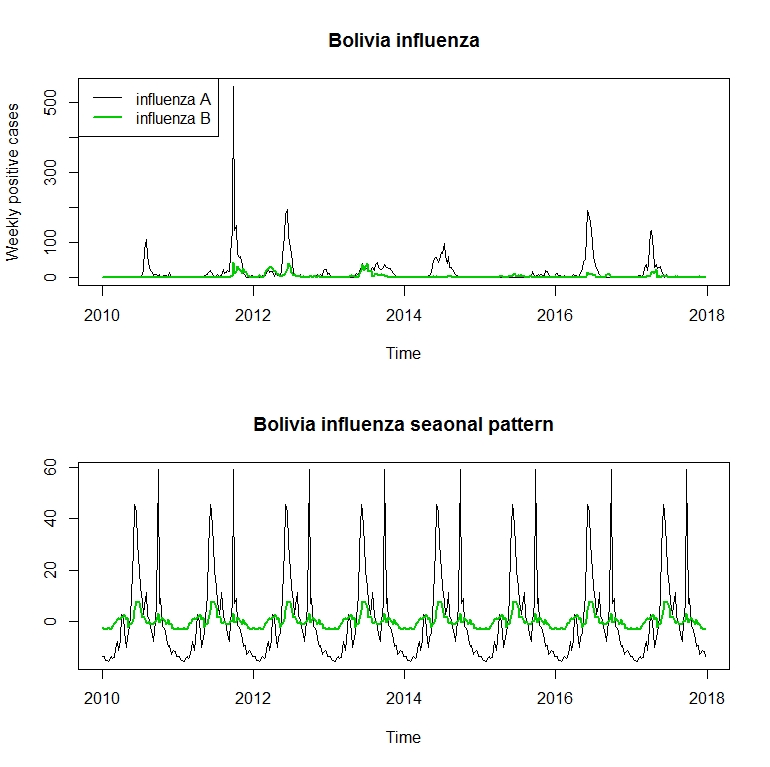


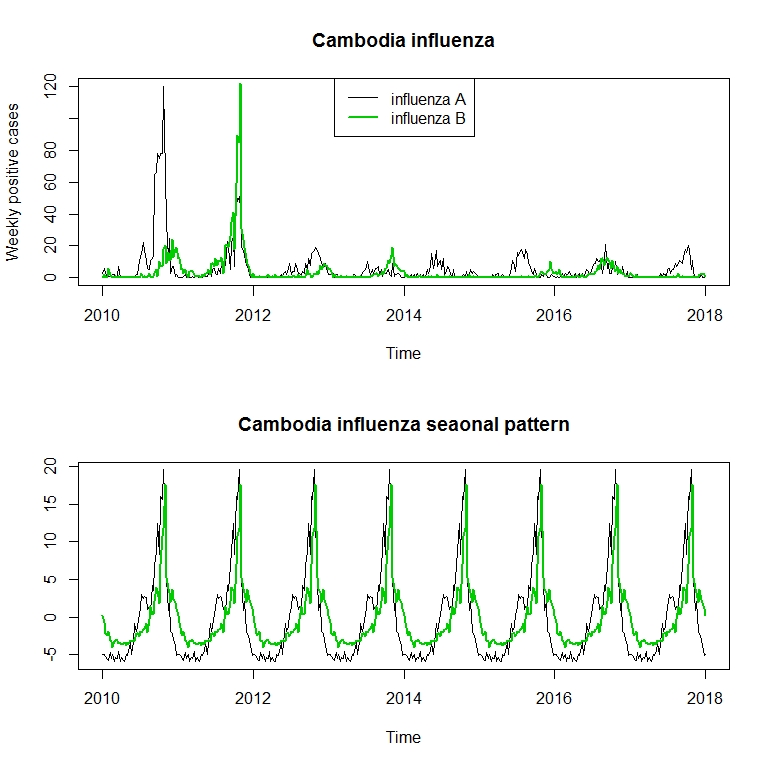


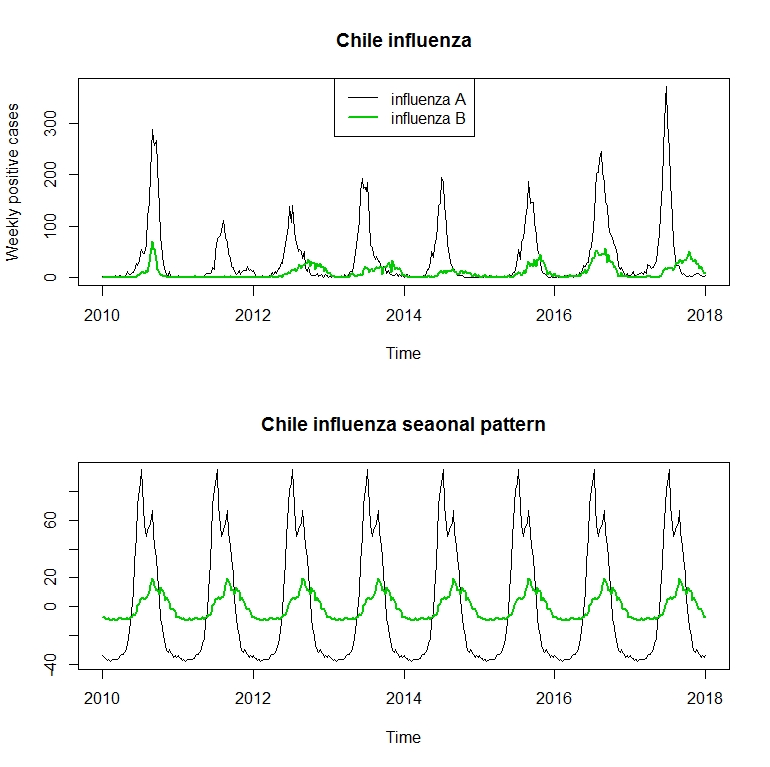


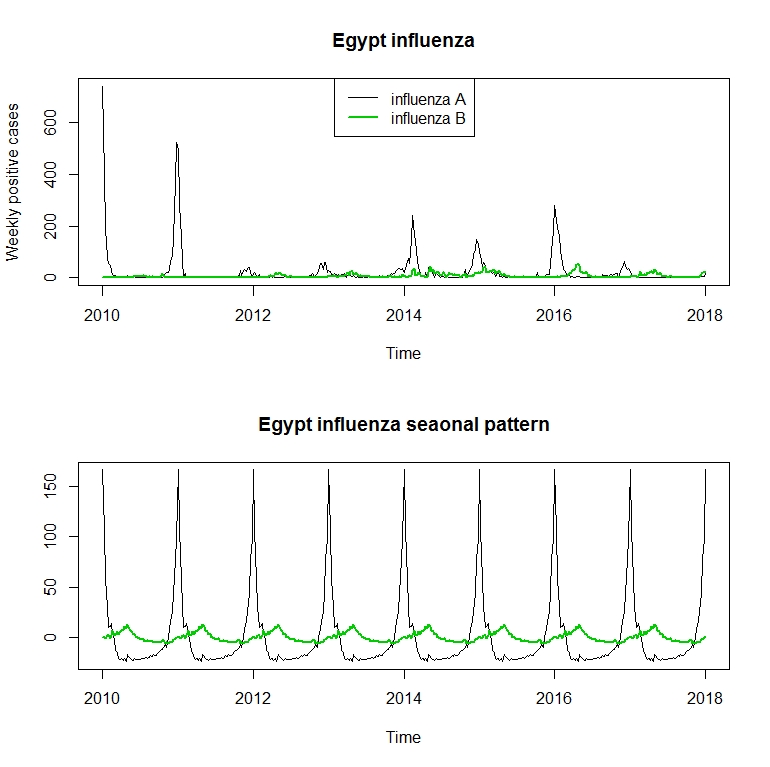


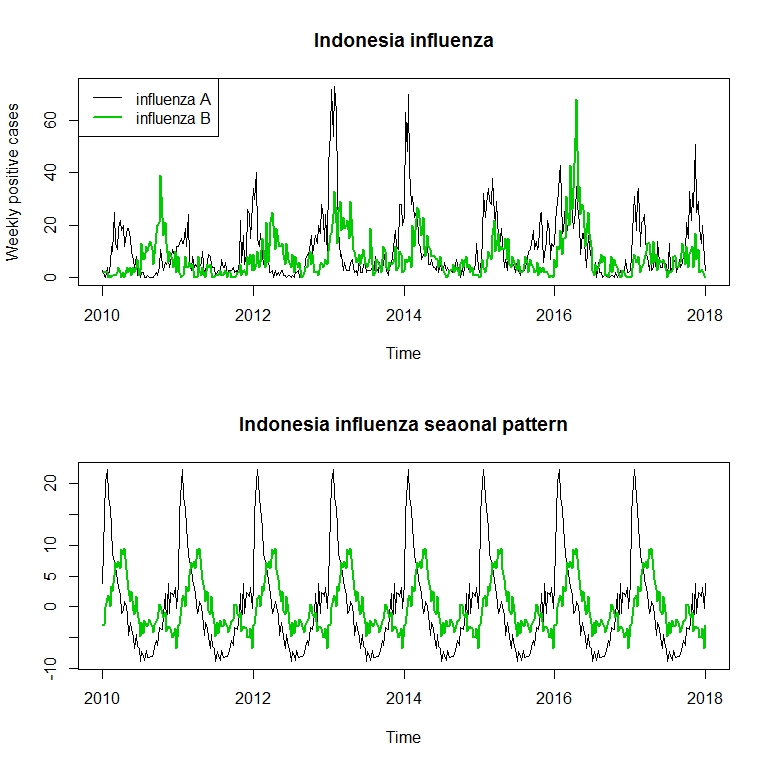


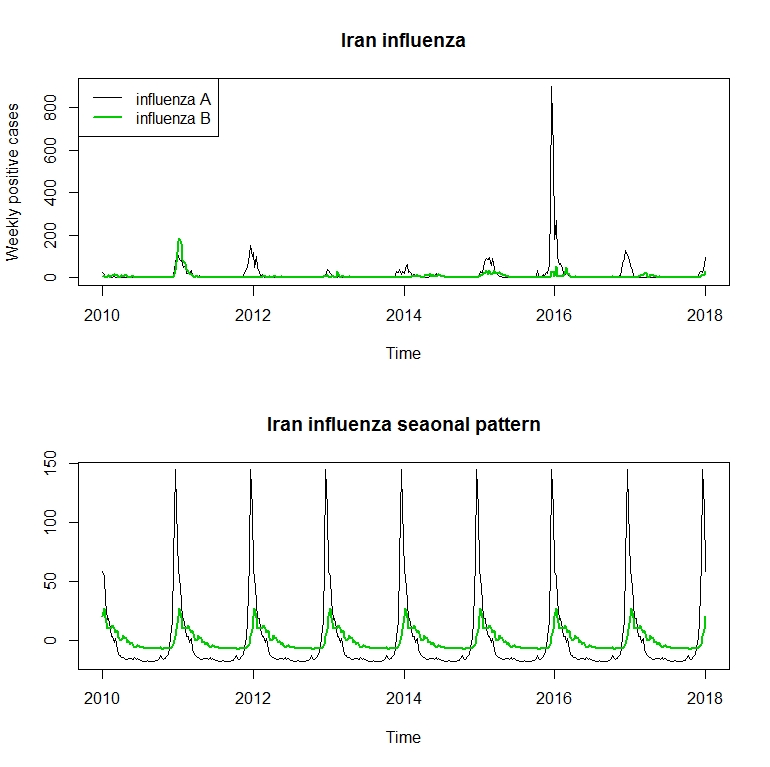


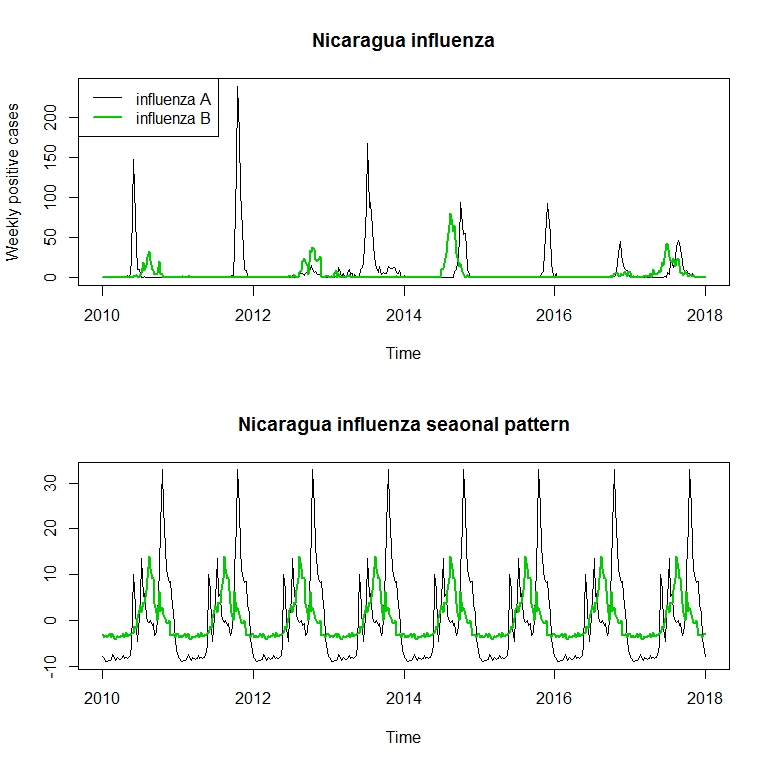


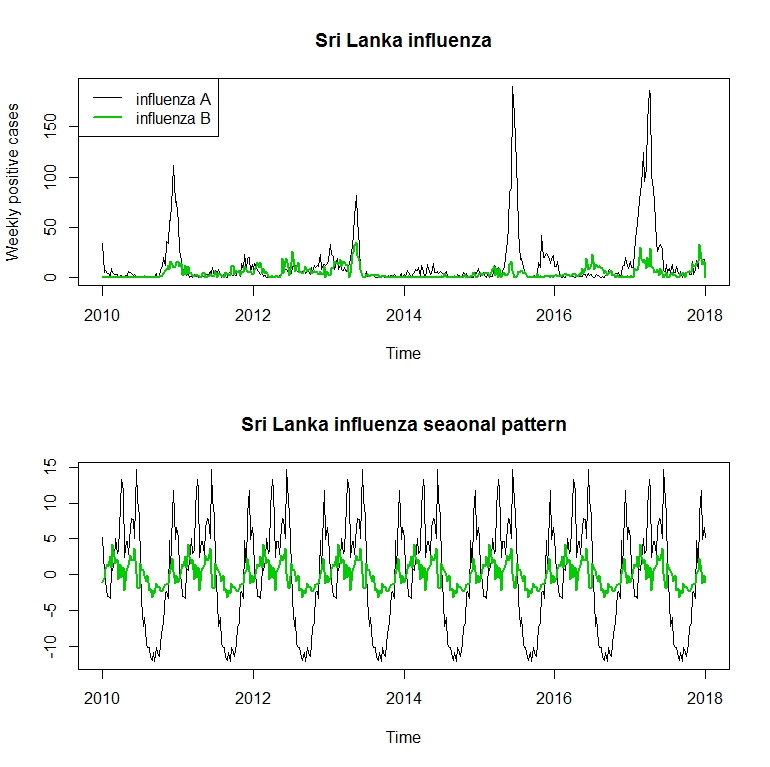

Supplement: Supplementary file 2 — Additional file 2: Figure S1. Seasonality patterns of influenza A and influenza B in countries of temperate climate and of tropical or subtropical climate, from 2010 to 2017. [file 40249_2019_618_MOESM2_ESM.docx]
